# DNA-SIP Reveals That *Syntrophaceae* Play an Important Role in Methanogenic Hexadecane Degradation

**DOI:** 10.1371/journal.pone.0066784

**Published:** 2013-07-01

**Authors:** Lei Cheng, Chen Ding, Qiang Li, Qiao He, Li-rong Dai, Hui Zhang

**Affiliations:** Key Laboratory of Development and Application of Rural Renewable Energy, Biogas Institute of Ministry of Agriculture, Chengdu, Sichuan, People’s Republic of China; Oak Ridge National Lab, United States of America

## Abstract

The methanogenic degradation of linear alkanes is a common process in oil-impacted environments. However, little is known about the key players involved in this process. Here, the hexadecane-degrading organisms in a methanogenic, hexadecane-degrading consortium designated M82 obtained from Shengli oilfield and maintained at 35°C for over 4 years, were identified by DNA-stable isotope probing with UL-^13^C-hexadecane, followed by density-resolved terminal restriction fragment length polymorphism (T-RFLP) analysis, cloning and phylogenetic analysis of 16S rRNA gene fragments. Compared to the fractions of the ^12^C treatment, the relative abundance of two phylotypes significantly increased in the heavy fractions of the ^13^C-hexadecane incubated microcosm. One belongs to a uncultured member of the bacterial family *Syntrophaceae*, which show 95–97% rRNA sequence identity with *Smithella propionica*, and the other is affiliated with *Methanoculleus receptaculi* (>99% sequence identity). The results of the present study prove the significant role of uncultured *Syntrophaceae* in degradation of hexadecane, probably through syntrophic interactions with hydrogenotrophic methanogens.

## Introduction

Crude oil is the world’s major source of energy and a critical raw material for many chemical products. However, spills of such contaminated compounds during exploration, transport and production may cause environmental pollution. On the other hand, large amounts of oil are trapped in most oil reservoirs even with state-of-the-art oil production techniques [1], [2]. Recently, the discovery of methanogenic degradation of hydrocarbons may offer an alternative route to clean up oil-contaminated environments and exploit difficult-to-use oil resources [3], [4].

Zengler et al. (1999) was the first to report methanogenic degradation of hexadecane, a representative aliphatic hydrocarbon of crude oil. Larter and colleagues inferred that anaerobic microbial activity may predominate in deep subsurface oil reservoirs [5], and deduced that the anaerobic degradation of hydrocarbons prevails in biodegraded oil reservoirs [6]. Recently, they proved that methanogenic degradation of hydrocarbons widely occurs in oil reservoirs and CO_2_ reduction is thought to be the dominant methane production pathway [7]. A large number of anaerobic microorganisms, including fermenting bacteria, nitrate-reducing bacteria, iron-reducing bacteria, sulphate-reducing bacteria and methanogens, have been detected in oil fields through culture-dependent and culture-independent approaches [8], [9]. The methanogenic communities degrading petroleum hydrocarbons have been reported to occur not only in petroleum reservoirs [7], [10], [11], [12], [13], but also in oil sands tailings ponds [14], [15], freshwater sediment [3] and oil-contaminated soils and sediments [16], [17], [18], suggesting that it is a common process in hydrocarbon-impacted environments. Thermodynamic analysis revealed the significant importance of syntrophic relationships between hydrocarbon-degraders and methanogenic archaea during methanogenic degradation of hydrocarbons [19]. Previous reports revealed that many uncultured phylotypes affiliated with syntrophic bacteria are present in petroleum hydrocarbon degrading methanogenic consortia [3], [7], [14], [20]. However, little is known about the actually degrading microorganisms under methanogenic conditions.

Stable isotope probing is a powerful method that directly links functional microorganisms to a specific biogeochemical process. This method is based on the principle that key players assimilate stable isotope-labeled substrates and convert them into cell biomass (nucleic acids, phospholipid fatty acids, or proteins), that can be detected through molecular ecological techniques [21]. Many pure isolates degrading aromatic and aliphatic hydrocarbons under anoxic conditions have been characterized using culture-dependent methods [22]. Furthermore, Several reports suggested that diverse uncultured clades are involved in the anaerobic degradation of aromatic hydrocarbons under sulphate-reducing [23], [24], nitrate-reducing [25], iron-reducing [26], and methanogenic conditions [18], [27]. However, limited studies have focused on long-chain alkane degraders under methanogenic conditions. Interestingly, recently an alkane-degrading sulphate-reducing bacterium was reported to degrade hexadecane to methane in co-culture with a hydrogenotrophic methanogen [28]. A methanogenic hexadecane-degrading consortium M82 was enriched from Shengli oilfield, and it was revealed that members of uncultured Waste Water of Evry 1 (WWE1), *Thermotogaceae*, and/or *Syntrophaceae* were the most common bacterial phylotypes [29]. However, who are major contributors responsible for the hexadecane degradation is still unclear. In the present study, a time-resolved DNA-SIP experiment using UL-^13^C-hexadecane was applied to this enriched consortium to unravel the key players involved in anaerobic hexadecane degradation and methane production.

## Materials and Methods

### SIP Incubation

Aliquots of 7 mL anoxic freshwater medium without sulphate and nitrate [30] were prepared and dispensed into 50 mL glass vials using Hungate anaerobic technique [31], in which Na_2_S.9H_2_O (0.3 g L^−1^), NaHCO_3_ (2.5 g L^−1^) and oil-contaminated soil extract (5 ml L^−1^) [32] were added. Resazurin (1 mg L^−1^) was used as a redox indicator, A hexadecane-degrading methanogenic consortium M82, maintained at 35°C in our laboratory for several years, which was obtained from Shengli oilfield, was selected for SIP incubation with 30% inoculum (v/v) [29]. A total of three sets of treatments were prepared: one set of microcosms was incubated by adding 1 mL of mixture of UL-^13^C-hexadecane (99 atom%, Sigma-Aldrich) and 2,2,4,4,6,8,8-heptamethylnonane (HMN) (v/v, 5: 1000) (13 replicates), the other set was amended with the same volume of unlabeled hexadecane and HMN (21 replicates). While the control group only received 1 mL HMN (17 replicates).The experiment was set up in a gas atmosphere of 80% N_2_ and 20% CO_2_ and statically incubated at 35°C in the dark.

### Process Measurements

The time course of methane production was measured using a gas chromatograph with a thermal conductivity detector as previously described [33]. The isotopic composition of methane was determined through a TraceGas system interfaced with a mass spectrometer (IsoPrime100, United Kingdom), briefly, gas samples (ca. 0.5 mL) withdrawn from the headspace of the culture vials were diluted and flushed into an IsoPrime trace gas pre-concentrator, CO_2_ and trace amount of water were removed through a catalyst (Ascarite+Mg(ClO_4_)_2_) and automated cold trap. The purified methane (ca. 0.7 µL) was converted into CO_2_ in a combustion furnace at a temperature of 1050°C. Helium (99.999%) was used as a carrier gas at a flow rate of 20 ml min^−1^. The carbon isotopic abundance of CO_2_ was reported relative to the Vienna PeeDee Belemnite.

One-way analysis of variance (ANOVA) for comparison of the difference between groups with or without hexadecane was accomplished using SPSS for windows 16.0 (The mean difference is significance at the 0.05 level).

### Nucleic Acid Extraction and Ultracentrifugation

Triplicate vials were sacrificed periodically for molecular analysis during the experiment (The ^13^C-hexadecane group: day 126, 166 and 218, The ^12^C-hexadecane group: day 126, 166, 198 and 218; The hexadecane-free group: day 126, 166, 198, and 218). Methanogenic cultures (4 mL) were shaken well before use and centrifuged at 17, 700 g for 4 min at 4°C, and stored at −80°C with the protection of RNAlater (Invitrogen, USA). Total genomic DNA was extracted via a bead-beating method [34], purified using the Promega Wizard DNA cleanup system (Promega, USA), and quantitatively determined with the Quant-iT™ PicoGreen® reagent (Invitrogen, USA). Genomic DNA (0.7–2 µg) was loaded onto a caesium trifluoroacetate (CsTFA) solution (GE, USA) with average density of 1.560±0.004 g mL^−1^ dissolved in GB buffer [35]. Isopycnic centrifugation was performed at 45,000 rpm for 40 h at 20°C with a Beckman 90 Ti rotor using an Optima™ L-80 XP ultracentrifuge (Beckman, German). Eighteen equal gradient fractions (ca. 500 µL) of density-separated DNA were collected using a syringe pump (Leifu, China), and buoyant density was measured using an AR200 digital refractometer (Reichert, USA). The fractionated DNA recovered in fractions was precipitated by adding two volumes of polyethylene glycol and 1 µL glycogen as previously described [21].

### Quantification and T-RFLP Analysis of rRNA Genes in the Density Gradient Fractions

Bacterial 16S rRNA genes in genomic DNA retrieved from the gradient fractions was quantified using a CFX96 touch™ real-time PCR detection system (Bio-Rad, USA) with a primer pair B519f/907r [36]. The PCR reaction mixtures (20 µL) consisted of 2 µL DNA, 7 µL ddH_2_0, 10 µL SsoFast™ EvaGreen supermix (Bio-Rad, USA), and 0.5 µL each of forward and reverse primers (5 pmol). The PCR cycle was carried out as follows: 94°C for 3 min, followed by 40 cycles of 94°C for 30 s, 54°C for 30 s, and 70°C for 30 s. Finally the melting curve was analyzed between 65 and 95°C in 0.5°C increment. Almost full-length 16S rRNA genes of *Escherichia coli* strain JM109 were diluted (10^5^–10^10^ copies mL^−1^) and used to construct a standard curve [37]. Quantitative PCR (qPCR) assays for *Syntrophaceae*, *Methanoculleus* and *Methanosaetaceae* were carried out using primer sets Syn826f/Syn1263r [20], 298f/586R [38] and Mst702f/Mst826r [39], respectively. The PCR reactions were performed as described above, but with a PCR annealing temperature of 55°C for *Syntrophaceae*, 58°C for *Methanoculleus* and *Methanosaetaceae*. Clone sequences of BM_12 (KC460267), A5_2 (HQ689161) and A5_7 (HQ689186) were also diluted (10^3^–10^9^ copies mL^−1^) and used to construct standard curves, respectively. For the T-RFLP analysis, the density-resolved DNA fractions were PCR amplified using the primer pairs B27f/B907r for bacteria and A109f/A934r for archaea [34], [40]. The 5′ end of primers B27f and A934r were labeled with 6-carboxyfluorescein. The PCR products were purified with TIAN Quick Midi Purification Kit (Tiangen, China), and then digested at 37°C using *Hae* III for bacterial DNA (TakaRa, Japan) and at 65°C using *Taq* I for archaeal DNA (TakaRa, Japan). The following procedure for T-RFLP analyses was performed using identical settings to previous report [29].

### Cloning, Sequencing and Phylogenetic Analysis

The PCR mixtures and procedures for cloning were identical to the aforementioned T-RFLP analysis, except the primers that were not fluorescently labeled. The PCR products were gel purified and cloned into *E. coli* competent cells JM109 (Takara, Japan) using pMD™ 19-T vector (Takara, Japan) according to the manufacturer’s instructions. Single colonies were picked and sequenced using a Genetic Analyzer (ABI, USA) [34].

The sequences were checked using the “Chimera check with Bellerophon” program of the greengene database [41] and aligned using the Clustal X software [42]. Distance matrices were calculated using the DNAdist software within the PHYLIP software package version 3.69 with the F84 correction [43]. The aligned sequences were grouped into operational taxonomic units using the farthest-neighbor clustering algorithm of the DOTUR software with a 97% threshold [44]. Diversity coverage of the constructed clone library was calculated using Good’s formula [45]. The representative clones from each OTUs were compared to RDP type species using the seqmatch program to determine the closest relatives [46]. Phylogenetic trees of the bacterial and archaeal 16S rRNA genes were created using the neighbor-joining method of Mega 5.1, the genetic distance matrix was estimated using maximum composite likelihood method [47]. Bootstrap values were calculated after 1,000 replications. The GenBank accession numbers of the 16S rRNA gene sequences generated in this study are JX088262 to JX088362 and JX473480 to JX473581.

## Results

### Hexadecane Degradation under Methanogenic Conditions

**Figure 1:**
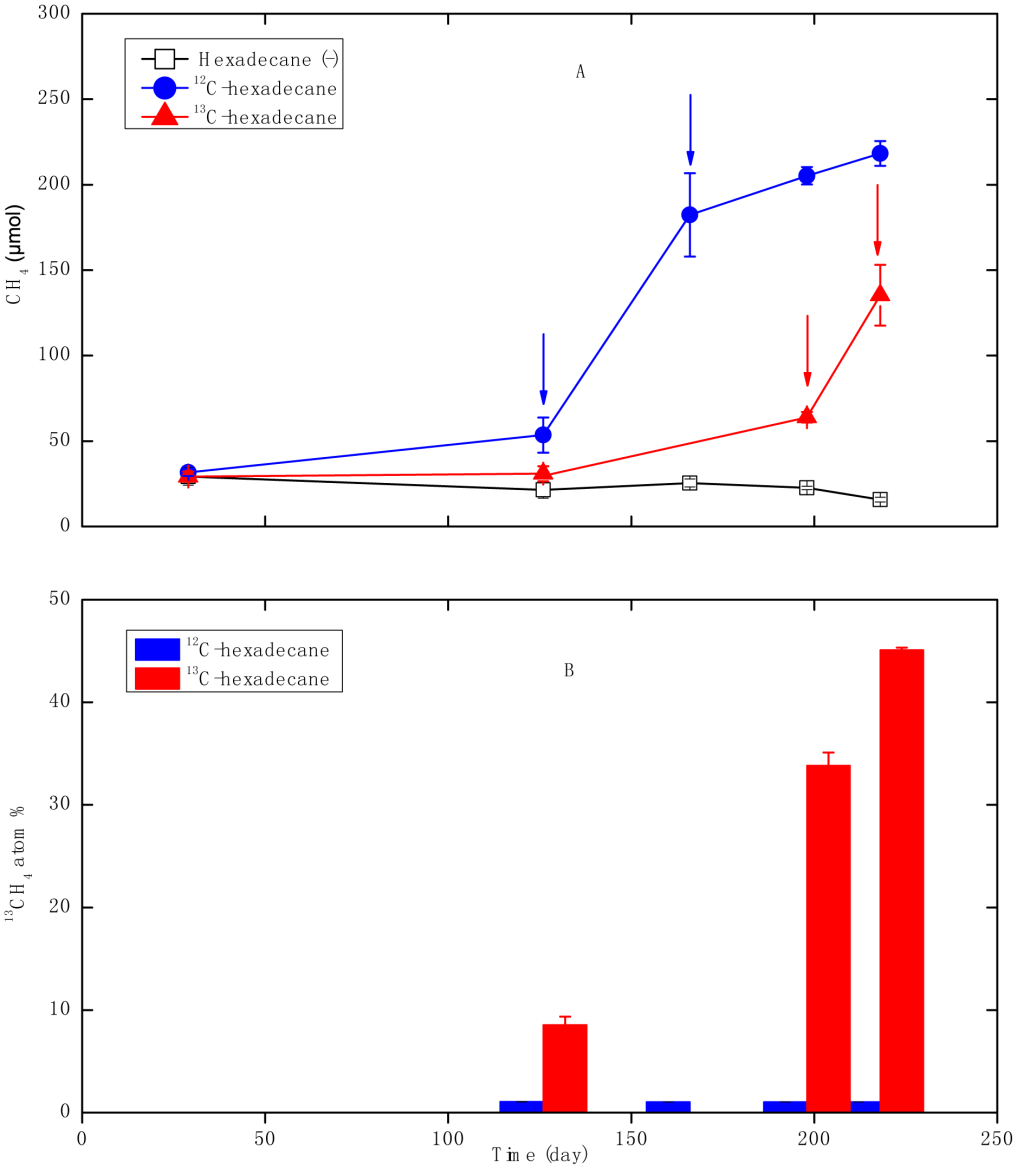
Time course of methane production and ^13^C/^12^C isotopic composition of methane of the consortia grown with hexadecane. A: Methane prodution; B:^ 13^C/^12^C isotopic composition of methane ^12^C-hexadecane: the consortium amended with unlabeled hexadecane; ^13^C-hexadecane: the consortium amended with 13C-labeled hexadecane; hexadecane (-): the controls without hexadecane addition, arrows indicate sampling points for the isopycnic centrifugation of genomic DNA. Error bars indicate standard deviations from three triplicates.

The ^12^C-hexadecane-degrading consortium began to accumulate methane after 126 days of incubation relative to the hexadecane free control incubations (p = 0.001), and generated a total of 218±7 µmol of methane after 218 days of incubation. By contrast, the control without hexadecane addition produced a negligible amount of methane that is only 16±1 µmol at day 218 (Fig. 1A). From 5 µL (ca. 17.1 µmol) hexadecane added, about 210 µmol of methane would be produced theoretically according to the stoichiometric conversion of hexadecane into methane and carbon dioxide as calculated by Symons and Buswell equation (Equation I), suggesting 96±3% of predicted methane was produced ((substrate-amended methane minus substrate-unamended methane)/theoretical methane). Compared to the group in which ^12^C-hexadecane was added, the enrichment cultures with ^13^C-hexadecane probably exhibited a lag of greater than 126 days before significant methane production was observed relative to hexadecane free control incubations (p = 0.06). It began to produce a substantial increase of methane production after 198 days of incubation (Fig. 1A), and generated about 120±18 µmol of methane at day 218 relative to the substrate-unamended control; corresponding to 57±9% theoretically predicted maximum possible methane production. The isotopic composition of ^13^CH_4_ generated from the ^13^C-hexadecane consortium increased up to 45.1±0.3% after 218 days of incubation (Fig. 1B), while the value retrieved from unlabeled hexadecane consortium didn’t exceed 1.051±0.001% during 218 days of incubation. These results confirmed the process of methanogenic degradation of hexadecane. Four replicates of enrichment cultures amended with ^13^C-hexadecane (total thirteen replicates) didn’t produce methane within *ca.* 200 days of incubation for unknown reasons.
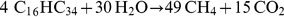
(1)


### Quantitative and Qualitative Analyses of Comparative SIP Gradients

As methane production progressed, several different vials were sacrificed for the molecular analysis at different time points (The ^13^C-hexadecane group: day 126, 166 and 218; the ^12^C-hexadecane group: day 126, 166, 198 and 218; the hexadecane-free group: day 126, 166, 198, and 218). T-RFLP analysis revealed that the bacterial communities was mainly composed of T-RFs 77, 160, 203, 207, 215 and 337 bp (Fig. S1A). T-RF 207 bp increased markedly over time in abundance in ^12^C- or ^13^C-hexadecane microcosms compared to the hexadecane-free group (Fig. S1A). The archaeal T-RFLP profiles of the 16S rRNA genes were mainly composed of four T-RFs of 186, 228, 284 and 495 bp in the hexadecane free, ^12^C-hexadecane, and ^13^C-hexadecane microcosms (Fig. S1B), the 186-bp T-RF increased over time in hexadecane addition group (Fig. S1B).

**Figure 2:**
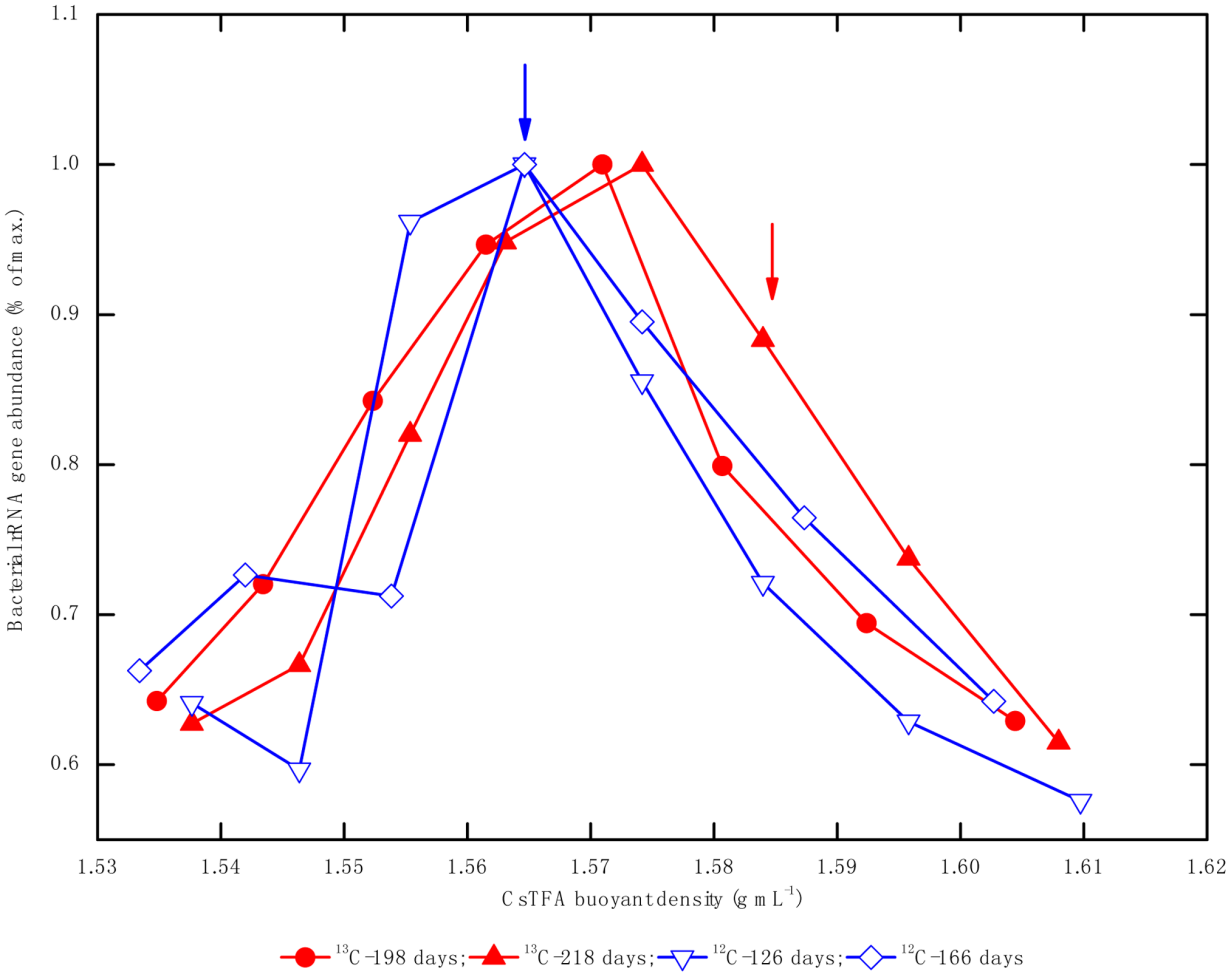
Relative abundance of bacterial 16S rRNA genes in the gradient fractions. ^12^C-hexadecane: the consortium amended with unlabeled hexadecane; ^13^C-hexadecane: the consortium amended with ^13^C-labeled hexadecane, arrows indicate sampling points of density fraction for construction of 16S rRNA gene clone libraries.

**Figure 3:**
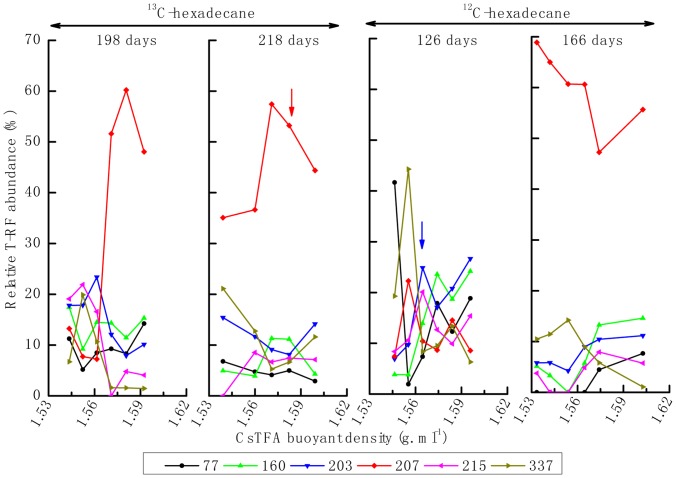
Relative abundance of bacterial T-RFs across different CsTFA buoyant densities.

**Figure 4:**
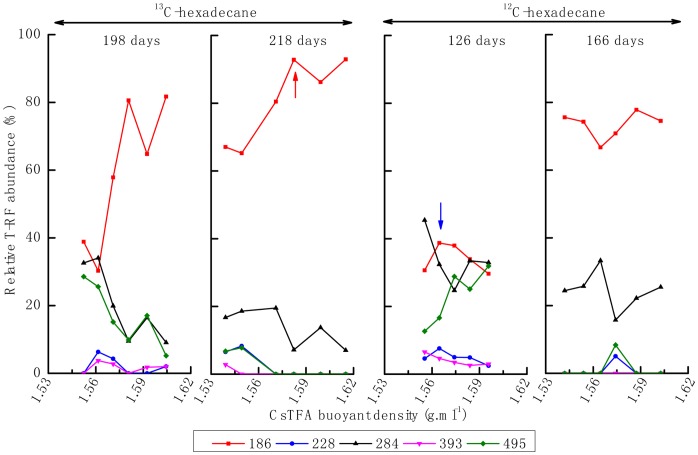
Relative abundance of archaeal T-RFs across different CsTFA buoyant densities.

For the difference of lag period between ^12^C- and ^13^C-hexadecane microcosms, the genomic DNA for isopycnic centrifugation was extracted from exponential phase of both enrichment cultures, not from the same day of incubation. qPCR analysis of the bacterial 16S rRNA gene copies in each individual fraction revealed that the peak of the bulk DNA, with ^12^C-hexadecane incubation at day 126 and a buoyant density of 1.565 g mL^−1^, was similar to that at day 166. However, a peak at 1.571 g mL^−1^ was detected at day 198 in the ^13^C-hexadecane consortium, shifted slightly to 1.574 g mL^−1^ after 218 days of incubation (Fig. 2). The relative abundances of bacterial and archaeal 16S rRNA genes from different gradient fraction were analyzed through T-RFLP fingerprinting. For the ^13^C-hexadecane incubation, bacterial T-RF 207 bp was significantly enriched in the “heavy” fractions of the density gradient after 198 and 218 days of incubation. By comparison, this fragment became dominant in all of the density fractions of ^12^C-hexadecane consortium from 126 to 166 days of incubation, and exhibits a decrease trend from light to heavy fractions of the density gradient. Other T-RFs, such as those with 77- and 160-bp, were slightly enriched at the beginning of the incubations (day 126) with ^12^C- and ^13^C-hexadecane, respectively (Fig. 3). The archaeal T-RF 186 bp dominated the heavy density fractions after successive incubations (day 198 and 218) in the ^13^C-hexadecane incubation (Fig. 4). On the contrary, this fragment became dominant in all of the observed fraction densities with similar abundance levels in ^12^C-hexadecane microcosms after 166 days of incubation.

### Phylogenetic Identification of the Labeled 16S rRNA Gene

**Figure 5:**
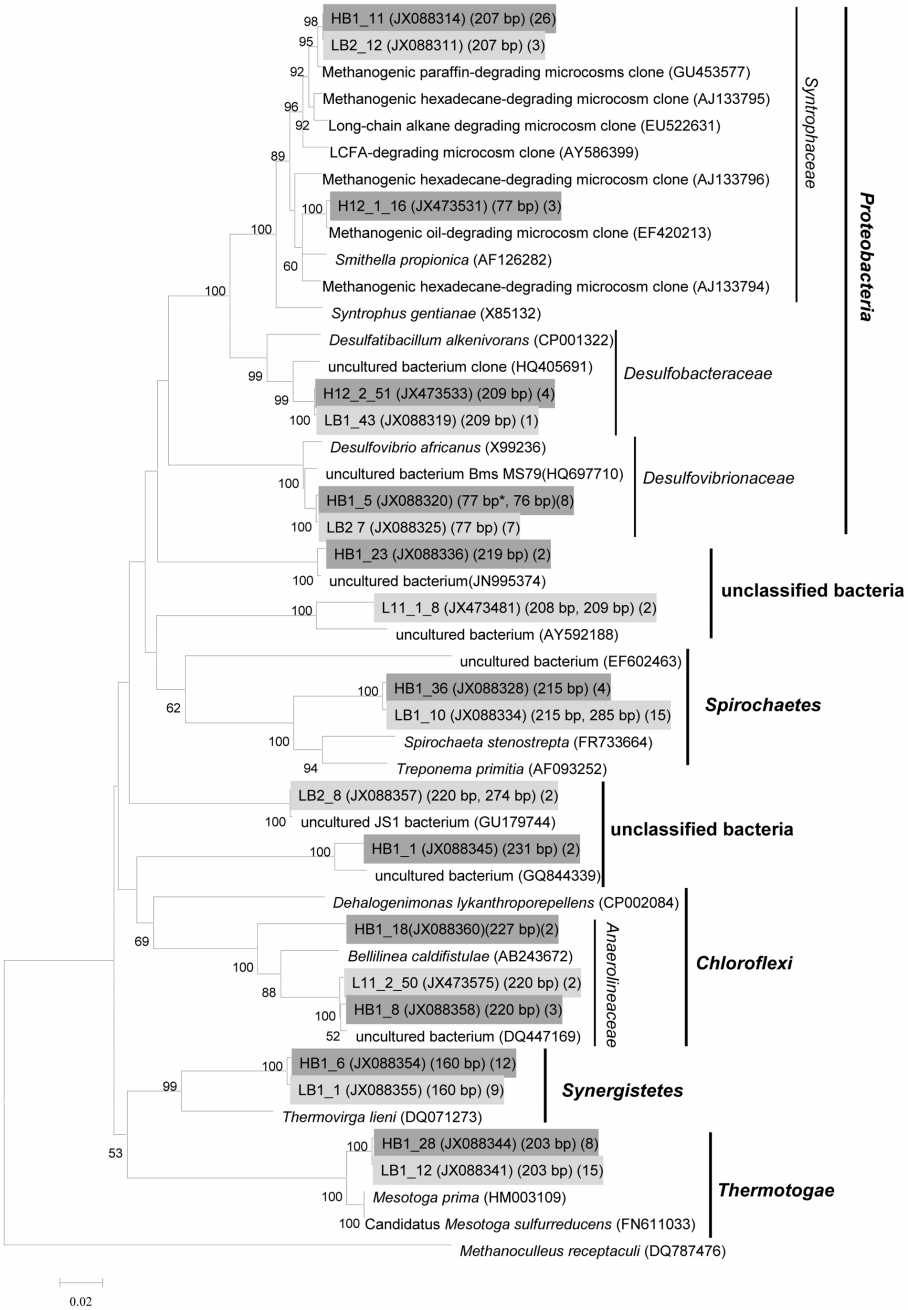
Phylogenetic tree based on bacterial 16S rRNA gene and related type strain and environmental clone sequences using the neighbor-joining method. The sequence of *Methanoculleus receptaculi* (DQ787476) was used as the outgroup. Scale bar, 2% estimated difference in nucleotide sequence. Numbers in the first parenthesis indicate GenBank number of type clone, the second represent the length of *in silico* T-RFs; and the thrid denote the clone number, the “light” clone sequences were labeled on a light gray background and the “heavy” were underlined on a dark gray background.

The *in vivo* T-RFs representing specific microbial lineages were characterized by cloning and sequencing of 16S rRNA genes. Two bacterial 16S rRNA gene clone libraries (total 154 clones) were generated from gradient fractions containing either “light” (unlabeled consortium on day 126, BD = 1.565 g mL^−1^) or “heavy” fraction (^13^C-labeled consortium on day 218, BD = 1.582 g ml^−1^) of nucleic acids as indicated by arrows in Fig. 2. The “heavy” bacterial clone library reached almost complete coverage (91.3%) based on the Good’s coverage estimator, and 75.3% was detected in the “light” clone library (Table 1, Fig. S2). Phylogenetic analysis revealed that over three-fourths of sequences were clustered into *Deltaproteobacteria, Thermotogaceae, Synergistaceae*, and *Spirochaetaceae* in both clone libraries (Fig. 5). Members of *Thermotogaceae* (20.5%), *Spirochaetaceae* (20.5%) *Synergistaceae* (14.8%) and *Desulfovibrionaceae* (9.6%) dominated the “light” community, while sequences affiliated with *Syntrophaceae* (35.8%), *Synergistaceae* (14.8%), *Thermotogaceae* (9.9%) dominated the “heavy” library (Table 1). *In silico* analysis of clone sequences revealed that the 207-bp T-RF represented members of *Syntrophaceae*, the 77-bp T-RF mainly represented members of *Desulfovibrionaceae,* 3 of 81 clones generated from “heavy” library belong to family *Syntrophaceae* also account for T-RF 77 bp. The *Spirochaetaceae*- and *Synergistaceae*-related members were mainly characterized by T-RF 215-bp and 160-bp, respectively. The uncultured WWE1 bacterium (Waste Water of Evry 1) was characterized by T-RF 337 bp. The *Thermotogaceae*-affiliated clones mainly represented a T-RF of 203 bp and dominated the “light” clone library (Table 1, Fig. 5).

**Table 1:** Phylogenetic affiliations and numbers of bacterial 16S rRNA sequences retrieved from clone libraries generated from different fractions.

Phylogenetic group&	*No*. of clones		*In silico*T-RF(bp)	Type clone	the closest type strains (similarity)
	^12^C-hexadecane (D126-1.565)	^13^C-hexadecane (D218-1.582)			
***Spirochaetes***					
unclassified *Spirochaetaceae* a	12	4	215	HB1_36	*Treponema primitia*; CP001843 (0.879)
***Proteobacteria***					
*Syntrophaceae*	3	26	207	HB1_11	*Smithella propionica*; AF126282 (0.956)
*Syntrophaceae*		3	77	H12_1_16	*Smithella propionica*; AF126282 (0.973)
*Desulfovibrionaceae* b	7	7	77	HB1_5	*Desulfovibrio africanus*; X99236 (0.981)
*Desulfobacteraceae*	1	4	209	H12_2_51	*Desulfatibacillum alkenivorans*; AY493562 (0.941)
***Synergistetes***					
*Synergistaceae*	9	12	160	HB1_6	*Thermovirga lieni*; DQ071273 (0.93)
***Thermotogae***					
*Thermotogaceae*	15	8	203	HB1_28	*Mesotoga sulfurreducens*; FN611033 (0.972)
***Chloroflexi***					
*Anaerolineaceae*	2	3	220	HB1_8	*Bellilinea caldifistulae*; AB243672 (0.924)
*Anaerolineaceae*		2	227	HB1_18	*Bellilinea caldifistulae*; AB243672 (0.912)
***Firmicutes***					
unclassified *Firmicutes*	2		220 (1), 274 (1)	LB2_8	*Moorella thermoacetica*; AY884087 (0.855)
unclassified bacteria	2		208 (1), 209 (1)	L11_1_8	*Desulfococcus multivorans*; AF418173 (0.819)
unclassified bacteria		2	219	HB1_23	*Calditerricola satsumensis*; AB250968 (0.847)
unclassified bacteria		2	231	HB1_1	*Phaselicystis flava*; EU545827 (0.793)
Total clones (Coverage)^c^	73 (75.3%)	81 (91.3%)			

Numbers in the parentheses of line “*In silico* T-RF (bp)” indicate clone numbers representing the corresponding T-RF.

& Clone sequences retrieved from “light” and “heavy” library was divided into OTU level in each row of the table. The OTUs containing one or no clone in both libraries were not shown in this table.

“^12^C-hexadecane (D126-1.565)” indicates that the clone library was constructed from the DNA fraction with a BD of 1.565 g. mL^−1^ of the unlabeled microcosm on day 126.

“^13^C-hexadecane (D218-1.582)” indicates that the clone library was constructed from the DNA fraction with a BD of 1.582 g. ml-1 of the ^13^C-labeled microcosm on day 218.

a Tree clones (account for T-RFs 285, 287 and 288 bp) retrieved from ^12^C-hexadecane (D126-1.565) representing for T-RFs 285, 287 and 288 bp are not shown in the table.

b One clone (account for T-RF 76 bp) retrieved from ^13^C-hexadecane (D218-1.582) accounting for T-RFs 76 is not shown in the tablec: The coverage was calculated based on Good formula [45], the 16S rRNA gene sequences were clustered into OTUs with 97% sequence identity.

**Figure 6:**
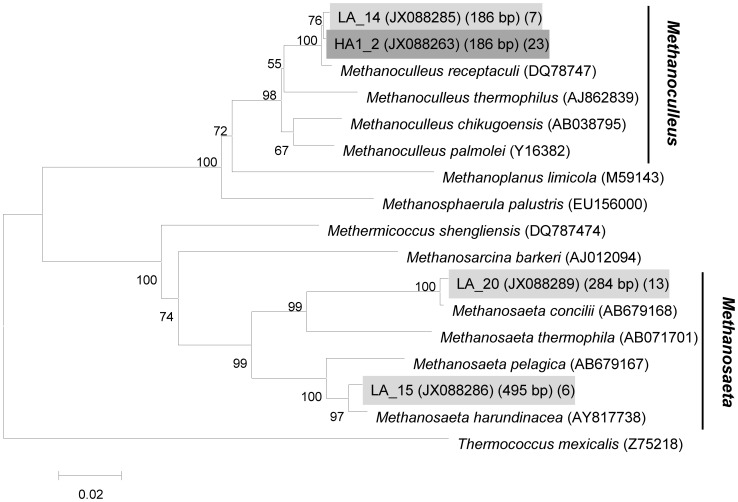
Phylogenetic tree based on the archaeal 16S rRNA gene and related type strain sequences using the neighbor-joining method. The bootstrap values are given at nodes when >50%. The sequence of *Thermococcus mexicalis* (Z75218) was used as the outgroup. Scale bar, 2% estimated difference in nucleotide sequence. Numbers in the first parenthesis indicate GenBank number of type clone, the second represent the length of *in silico* T-RFs; and the thrid denote the clone number, the “light” clone sequences were labeled on a light gray background and the “heavy” were underlined on a dark gray background.

Two archaeal 16S rRNA gene clone libraries (49 clones) were also retrieved from the “light” and “heavy” fractions as indicated by arrows in Fig. 2, respectively. All of the archaeal sequences belonged to the phylum *Euryarchaeota* (Table 2, Fig. 6), In the “light” clone library, the majority of archaeal clones (73.1%) were clustered into acetoclastic *Methanosaeta*, and showed quite high 16S rRNA sequence identity (>99%) to *Methanosaeta concilii* (T-RF 284 bp) or *Methanosaeta harundinacea* (T-RF 495 bp). The remaining clones showed 99% 16S rRNA sequence identity to *Methanoculleus receptaculi*, represented by a T-RF of 186 bp. By contrast, all the clones in the “heavy” library belonged to members of *M. receptaculi* (>99%). No clones accounting for 228- and 393-bp T-RF were detected in both microcosms, but these two fragments may be assigned to *Methanosaetaceae* and *Methanomicrobiaceae* respectively, based on our previous study using the same inoculum [29].

**Table 2:** Phylogenetic affiliations and numbers of archaeal 16S rRNA sequences retrieved from clone libraries generated from different fractions.

Phylogenetic group	*No.* of clones		*In silico* T-RF (bp)	Type clone	the closest type strains (similarity)
	^12^C-hexadecane (D126-1.565)	^13^C-hexadecane (D218-1.582)			
*Methanoculleus*	7	23	186	LA_14	*Methanoculleus receptaculi*; DQ787476 (0.995)
*Methanosaeta*	13		284	LA_20	*Methanosaeta concilii*; X51423 (0.994)
	6		495	LA_15	*Methanosaeta harundinacea*; AY817738 (0.990)
Total clones (coverage)^a^	26 (nd)	23 (nd)			

a the value of coverage index was not generated for OTU with one sequence was not detected.

qPCR analysis across all fractions revealed that the peak of 16S rRNA gene of *Syntrophaceae* was primarily detected in the fraction of 1.565 g mL^−1^ in ^12^C-hexadecane incubation, shifted to fractions above 1.571 g mL^−1^ in ^13^C-hexadecane incubation (Fig. S3A). *Methanoculleus* and *Methanosaetaceae* also shifted from unlabeled to ^13^C-labeled microcosms (Figs. S3B and S3C). A tailing of DNA indicative of *Methanoculleus* into the heavy gradient fraction (1.574 g mL^−1^) of ^12^C-hexadecane microcosm at day 166 was also observed (Fig. S3C). Further analysis revealed that Log 16S rRNA gene of *Syntrophaceae* increased by 1.13±0.69 log units in ^12^C-hexadecane microcosm (per mL) from day 126 to 218 (Fig. S4A), the average increase in abundance of *Methanoculleus* was 1.53±0.49 log units, which is higher than *Methanosaetaceae* (0.62±0.41 log units) ((Figs. S4B and S4C).

## Discussion

To our knowledge, the present study is the first to characterize the key players involved in the methanogenic degradation of long chain n-alkane using DNA-based SIP. ^13^C-hexadecane degradation to methane was retarded compared to ^12^C-hexadecane (Fig. 1A), which was also reported previously [3]. However, Fowler *et al*. reported similar growth curves in a methanogenic consortium degrading ^12^C-toluene and ^13^C-toluene, respectively [48]. One may speculate that there are differences between organisms: some may be more sensitive and some more “tolerant” towards non-natural isotope contents in their substrates (personal communications with Dr. Friedrich Widdel). Members of *Syntrophaceae* accounting for bacterial T-RF 207 bp may have a more active role in hexadecane degradation as the majority of ^13^C-labeled carbon was incorporated into DNA from this phylotype. This was confirmed by the fact that the 207-bp T-RF became predominant in the ^12^C-hexadecane consortium at the later logarithmic growth phase. The closest cultured representative of the *Syntrophaceae* sequences (95%–97% 16S rRNA sequence identity) retrieved in this study is *Smithella propionica* LYP, a syntrophic bacterium isolated from an anaerobic digester, which dismutated propionate to acetate and butyrate [49], [50]. *Syntrophaceae*-affiliated clones have been detected in a vast number of methanogenic consortia degrading alkanes and petroleum hydrocarbon [3], [7], [12], [14], [15], [16], [51], suggesting their potential ecophysiological role in the process of hydrocarbon degradation under methanogenic conditions. Gray *et al*. revealed a significant positive correlation between *Syntrophaceae*-affiliated clones and methane production from a petroleum hydrocarbon-degrading consortium, and proposed that the phylotype played an important role in complete oxidation of crude oil alkanes to acetate and/or hydrogen [20]. In this study, we further identified a novel uncultured member of *Syntrophaceae* that probably played a key role in hexadecane degradation under methanogentic conditions using DNA-SIP.

Other bacterial T-RFs such as those of 77, 160, 203, 215 and 337 bp were also detected in the ^12^C- and ^13^C-hexadecane enrichment cultures. Dissimilatory sulfate-reducing bacterium *Desulfovibrio africanus* DSM 2603^T^ shows 98% 16S rRNA sequence identity to type clone HB1_5 (accounts for T-RF 77 bp), which exhibits syntrophic oxidation of organic acids under sulphate-limited conditions in co-culture with methanogens, and could use H_2_ as an electron donor and assimilate acetate and CO_2_ as carbon sources [52]. The *Desulfovibrio-*like bacterium ACE-8 (GenBank *No.* JX477133) was isolated from this consortium in our laboratory. No growth occurred after nine months of incubation when co-cultured with *M. recepatculi* ZC-2 using hexadecane as a sole substrate at 35°C (data not shown). This implies that the *Desulfovibrio-*like organism may metabolize intermediates generated from hexadecane degradation. However, a few clones accounting for T-RF 77 bp also belong to *Syntrophaceae*, and the utilization of hexadecane for growth by this organism can’t be excluded.

Clones (type clone HB1_6) clustered in *Synergistaceae* shared a T-RF of 160 bp, and has 93% 16S rRNA gene sequence identity to *Thermovirga lienii*, which degrades proteinaceous substrates, amino acids, and a limited range of organic acids, but not sugars, fatty acids or alcohols. It produces ethanol, acetate, propionate, isovalerate/2-methylbutyrate, H_2_ and CO_2_ [53]. The *Thermotogaceae*-related member (type clone HB1_28) representing a T-RF of 203 bp, shares 98% sequence similarity to *Mesotoga prima* and Candidatus *Mesotoga sulfurreducens* [54], [55], which is a novel phylogenetic lineage in the order *Thermotogales* and grows on sugars and some proteinaceous substrates with acetate as a major fermentation product. The *Spirochaetaceae*-affiliated member (mainly accounts for T-RF 215 bp) exhibits 88% 16S rRNA sequence identity with the homoacetogenic *Treponema primitia* ZAS-2^T^ [56], which has the physiological potential to utilize H_2_ and CO_2_ as alternative energy sources, and possibly possesses the capacity to compete with hydrogenotrophic methanogens. Uncultured WWE1 bacterium (T-RF 337 bp) always predominated in the methanogenic hexadecane-degrading consortium during transfer incubations [29], became less abundant after HMN addition as a carrier of hexadecane. Genome analysis of Candidatus *Cloacamonas acidaminovorans*, an uncultured representative bacterium of the WWE1 bacterium, revealed the potential of oxidation of propionate into acetate and carbon dioxide [57]. From the wide distribution but low abundance of members of *Desulfovibrionaceae*, *Thermotogaceae*, *Spirochaetaceae*, *Synergistaceae* and uncultured WWE1 bacterium in this study, we proposed that the ecophysiological role of these phylotypes may be associated with the utilization of intermediates from hexadecane degradation and even dead biomass, and were by far not as important as the members of the *Syntrophaceae* counterparts detected in our SIP incubation. However, their ecological significance in oil-impacted environments should be further investigated due to their wide distribution [9], [10], [58], [59], [60]. The *Desulfobacteraceae*-affiliated member (4 in 81 clones) detected in “heavy” clone library (Table 1), was most closely related to *Desulfatibacillum alkenivorans*, a known alkane-degrader under sulphate-reducing condition, which could degrade hexadecane in co-culture with hydrogenotrophic methanogen [28]. However, the corresponding T-RF 209 bp was not detected with high abundance (<1%) through T-RFLP analysis during SIP incubation, suggesting their minor role in hexadecane conversion.

The archaeal community with ^13^C-labeled rRNA genes in heavy fractions only consisted of *M. receptaculi*-affiliated organisms (T-RF 186 bp). *M. receptaculi*, which was isolated from Shengli oilfield, is a hydrogenotrophic methanogen that uses hydrogen and carbon dioxide as energy and carbon sources, grows best at 55°C, and demonstrates methanogenic activity under mesophilic conditions [33]. qPCR assay also confirmed that the increase of *Methanoculles* species is much higher than member s of *Methanosaetaceae* (Figs. S4B and 4C). The results demonstrate that, the principal pathway of methane production may be mainly through carbon dioxide reduction in our incubation of hexadecane degradation. However, a certain abundance of *Methanosaeta* species in hexadecane incubation may suggest that acetoclastic methanogens use small amounts of acetate from ^13^C-hexadecane. This is consistent with the findings of Gray *et al*., 2011 who demonstrated that both acetoclastic and CO_2_-reducing methanogens were enriched during methanogenic degradation of crude oil alkanes, but CO_2_-reducing methanogens were enriched to a greater extent. Moreover this is what would be expected if syntrophic acetate oxidation was an important process in methanogenic alkane degradation as previously suggested [20] This is also consistent with modeling of gas isotopic composition in biodegraded petroleum reservoirs that suggested that most, but not all methanogenic hydrocarbon degradation was channeled through the CO_2_ reduction pathway [7].

Methanogenic degradation of complex compounds is a common process under electron-acceptor limited conditions, and the ability of bacteria and methanogenic archaea to degrade hydrocarbons collectively via syntrophic cooperation is definitely necessary [19]. In the present study, so-far uncultured members of *Syntrophaceae* were shown to play a vital role in hexadecane degradation, mainly coupled with hydrogenotrophic *M. receptaculi*-related archaea.

## Supporting Information

Figure S1 **The T-RFLP profiles at different days of incubation.** A: bacterial domain; B: archaeal domain. ^12^C-hexadecane: the consortium amended with unlabeled hexadecane; ^13^C-hexadecane: the consortium amended with ^13^C-labeled hexadecane; hexadecane (−): the controls without hexadecane addition, error bars indicate standard deviations from three triplicates.
(TIF)

Figure S2 **Rarefaction curves constructed from two bacterial 16S rRNA gene libraries based on OTU cutoff of equal or higher 97%.** ^12^C-hexadecane: the consortium amended with unlabeled hexadecane; ^13^C-hexadecane: the consortium amended with ^13^C-labeled hexadecane.
(TIF)

Figure S3 **Relative abundance of 16S rRNA genes in the gradient fractions of enrichment cultures amended with ^12^C- and ^13^C-hexadecane.** A: *Syntrophaceae*, B: *Methanoculleus* and C: *Methanosaetaceae*. ^13^C-198 days (•): Genomic DNA retrieved from 13C-hexadecane microcosm at day 198; ^13^C-218 days (▴): Genomic DNA retrieved from 13C-hexadecane microcosm at day 218; ^12^C-126 days (Δ): Genomic DNA retrieved from 12C-hexadecane microcosm at day 126; ^12^C-166 days (◊): Genomic DNA retrieved from 12C-hexadecane microcosm at day 166. Error bars indicate standard deviations from three triplicates.
(TIF)

Figure S4 **Time course of log 16s rRNA gene abundance in SIP microcosms (log gene abundance per mL).** A: *Syntrophaceae*; B: *Methanoculleus*; C: *Methanosaetaceae*. ^13^C-198 days (•): Genomic DNA retrieved from 13C-hexadecane microcosm at day 198; ^13^C-218 days (▴): Genomic DNA retrieved from ^13^C-hexadecane microcosm at day 218; ^12^C-126 days (△): Genomic DNA retrieved from ^12^C-hexadecane microcosm at day 126; ^12^C-166 days (◊): Genomic DNA retrieved from ^12^C-hexadecane microcosm at day 166. We purified genomic DNA by washing twice the filtration column (first: 50 µL; second: 30 µL) when using Promega Wizard DNA cleanup system (Promega, USA). The entire first washed DNA sampled from ^13^C-hexadecane microcosm at day 126 was used for isopycnic centrifugation, and the second washed DNA was used for T-RFLP analysis. So the data for quantitative analysis of gene copies of ^13^C-hexadecane microcosm at day 126 was not shown, error bars indicate standard deviations from three triplicates.
(TIF)
