# Supplementary figures and images for: DNA-SIP Reveals That Syntrophaceae Play an Important Role in Methanogenic Hexadecane Degradation
Source: PLoS One. 2013 Jul 1;8(7):e66784. doi: 10.1371/journal.pone.0066784 (PMC3698093; doi:10.1371/journal.pone.0066784)

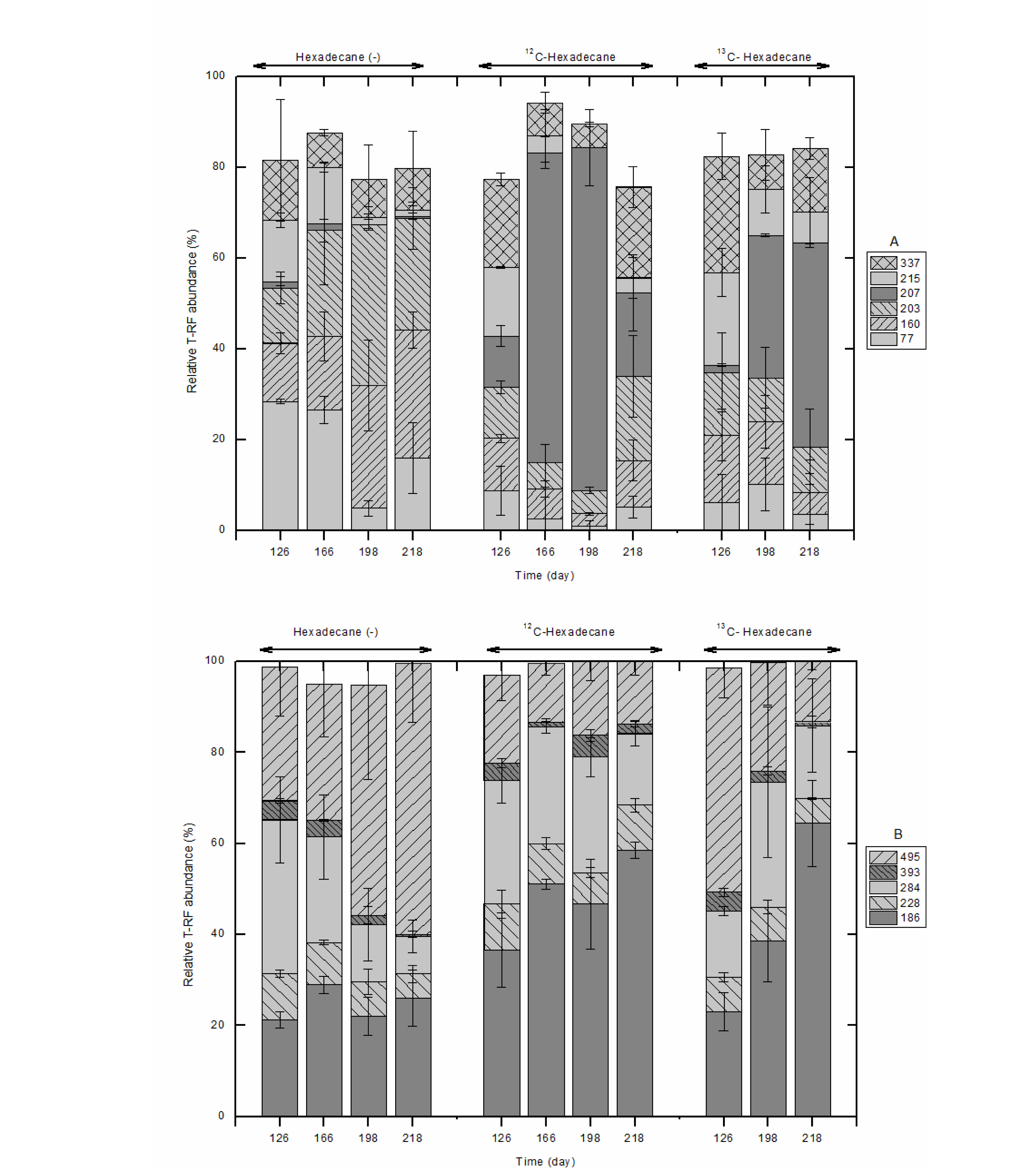

Supplement: Figure S1 — The T-RFLP profiles at different days of incubation. A: bacterial domain; B: archaeal domain. 12C-hexadecane: the consortium amended with unlabeled hexadecane; 13C-hexadecane: the consortium amended with 13C-labeled hexadecane; hexadecane (−): the controls without hexadecane addition, error bars indicate standard deviations from three triplicates. (TIF) [file pone.0066784.s001.tif]

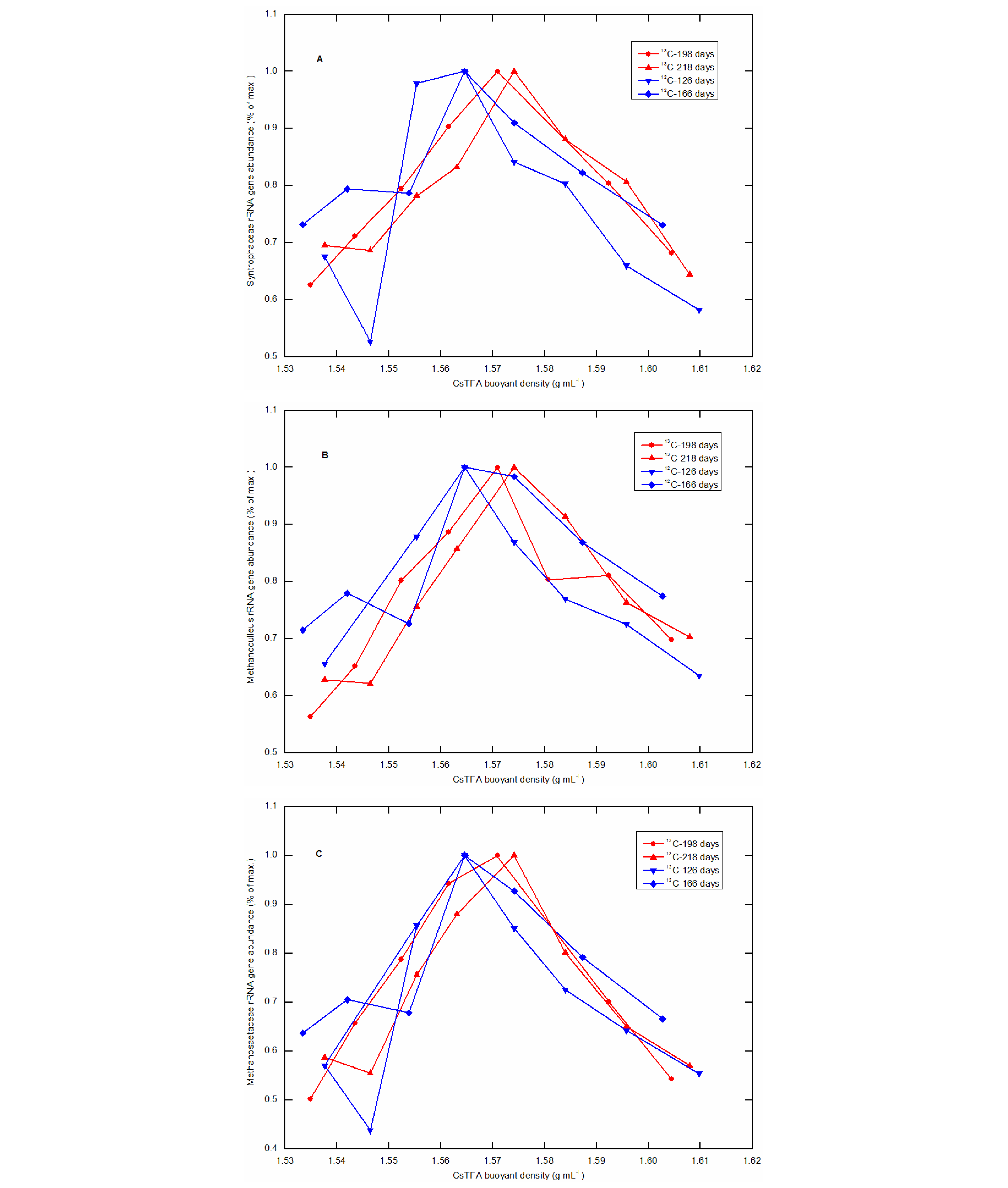

Supplement: Figure S3 — Relative abundance of 16S rRNA genes in the gradient fractions of enrichment cultures amended with 12C- and 13C-hexadecane. A: Syntrophaceae, B: Methanoculleus and C: Methanosaetaceae. 13C-198 days (•): Genomic DNA retrieved from 13C-hexadecane microcosm at day 198; 13C-218 days (▴): Genomic DNA retrieved from 13C-hexadecane microcosm at day 218; 12C-126 days (Δ): Genomic DNA retrieved from 12C-hexadecane microcosm at day 126; 12C-166 days (◊): Genomic DNA retrieved from 12C-hexadecane microcosm at day 166. Error bars indicate standard deviations from three triplicates. (TIF) [file pone.0066784.s003.tif]

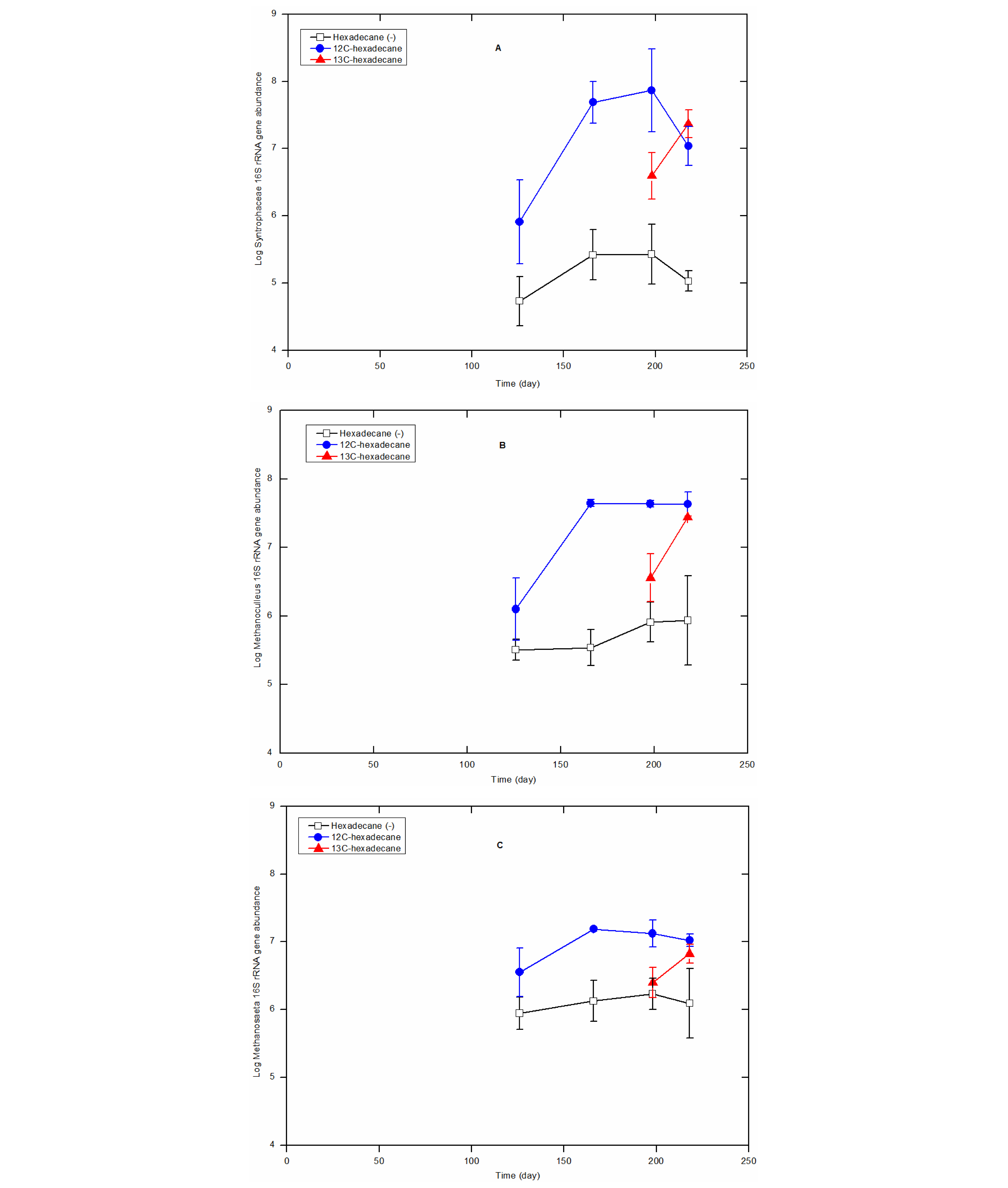

Supplement: Figure S4 — Time course of log 16s rRNA gene abundance in SIP microcosms (log gene abundance per mL). A: Syntrophaceae; B: Methanoculleus; C: Methanosaetaceae. 13C-198 days (•): Genomic DNA retrieved from 13C-hexadecane microcosm at day 198; 13C-218 days (▴): Genomic DNA retrieved from 13C-hexadecane microcosm at day 218; 12C-126 days (△): Genomic DNA retrieved from 12C-hexadecane microcosm at day 126; 12C-166 days (◊): Genomic DNA retrieved from 12C-hexadecane microcosm at day 166. We purified genomic DNA by washing twice the filtration column (first: 50 µL; second: 30 µL) when using Promega Wizard DNA cleanup system (Promega, USA). The entire first washed DNA sampled from 13C-hexadecane microcosm at day 126 was used for isopycnic centrifugation, and the second washed DNA was used for T-RFLP analysis. So the data for quantitative analysis of gene copies of 13C-hexadecane microcosm at day 126 was not shown, error bars indicate standard deviations from three triplicates. (TIF) [file pone.0066784.s004.tif]
